# Factors Affecting Binocular Sensorial Function in Accommodative Esotropia

**DOI:** 10.14744/bej.2021.77598

**Published:** 2021-09-27

**Authors:** Sezin Akca Bayar, Zeynep Kayaarasi Ozturker, Burak Ulas, Sibel Oto, Aysel Pelit, Seval Akgun

**Affiliations:** 1. Department of Ophthalmology, Baskent University, Ankara Hospital, Ankara, Turkey; 2. Department of Ophthalmology, Baskent University, Istanbul Hospital, İstanbul, Turkey; 3. Department of Ophthalmology, Baskent University, Adana Hospital, Adana, Turkey

**Keywords:** Accommodative esotropia, fusion, stereopsis

## Abstract

**Objectives:**

This study was an assessment of factors related to the development and maintenance of binocular sensory function after successful alignment of accommodative esotropia (AE).

**Methods:**

A total of 107 patients aged <12 years with ≥6 months follow-up were included in the study. The variables of age at onset of deviation, duration of deviation before treatment, the amount of uncorrected distance and near deviation, hyperopia, anisometropia, and accommodative convergence to accommodation ratio (AC/A) were evaluated.

**Results:**

The study patients had a mean age of 4.9±2.5 years and a mean length of follow-up of 34.3±28 months. Anisometropia was identified in 26.1% of the participants. Anisometropic patients had a greater degree of hyperopia (mean: 5.02±2.07 D) than the patients without (p>0.05). Amblyopia was seen in 25% of the patients with anisometropia, and in 19% of those without (p<0.05). The binocular visual function of the 2 groups was not significantly different (p>0.05). The age at onset of deviation and the duration of deviation did not affect the final outcome (p>0.05 for all). The degree of initial uncorrected distance deviation had a significant effect on the development of amblyopia, fusion, and contoured stereopsis (p<0.05 for all), while the degree of near deviation without correction had a significant effect only on contoured stereopsis.

**Conclusion:**

The degree of uncorrected distance and near deviation had a negative impact on binocular vision and stereopsis in AE. The presence of anisometropia, age at onset of deviation, duration of deviation before treatment, high hyperopia, and high AC/A did not pose a significant risk for impaired binocular function.

## Introduction

Accommodative esotropia (AE) is a convergent deviation of the eyes associated with activation of accommodative reflex (1). Refractive AE is defined as an esotropia that is restored to orthotropia at all fixation distances and in all gaze positions by optical correction of underlying hypermetropic refractive error (RE) (1, 2). AE typically occurs between the ages of 18 and 48 months following substantial maturation of binocular vision. In general, the prognosis for restoring normal binocular function in refractive AE is excellent if normal binocular vision occurred before the onset of the deviation. Early treatment helps prevent permanent loss of stereopsis and provides good binocular vision (3). Despite the relatively late onset, many children do not regain normal binocular vision even after successful realignment with spectacles, monocular occlusion, or surgery (4). One hypothesis to explain this situation was that foveal fusion mechanisms were permanently impaired during the deviation period. According to this assumption, irreversible defect of stereopsis can be preventable if the patients use correcting glasses at an early stage during intermittent esotropia or within 3 months after the onset of constant deviation (4-10).

Recent studies suggest that specific risk factors such as duration of constant misalignment, age of onset, high AC/A, hypermetropia, and anisometropia may play a critical role in binocular sensorial function of patients with AE (4-7, 9-16). In this study, we aimed to investigate the causative factors for abnormal binocular vision in patients with AE following successful refractive correction.

## Methods

This study was approved by Baskent University Institutional Review Board (Project no:KA06/301). All authors confirmed that the study and data collection conformed to all local laws and were compliant with the principles of the Declaration of Helsinki.

Retrospective analysis revealed 139 patients diagnosed with AE who had a regular follow-up for 6 months or longer at Baskent University Hospital, Department of Ophthalmology. Eligibility criteria included an esodeviation of <8 prism diopter (PD) with full cycloplegic refractive correction on the first examination and during the follow-up period. Exclusion criteria included the age of more than 12 years at the time of initial examination, a follow-up period of <6 months, amblyopia due to organic disorders, history of developmental delay or severe neurological conditions, and partial or decompensated AE.

Following a file search for patient data, we have prospectively completed additional patient data at each visit. Age of onset and duration of eye misalignment before treatment, previous occlusion therapy, or strabismus surgery were also obtained from the parents. If the onset of deviation was not clear, old photographs were evaluated.

All patients underwent full ophthalmic and orthoptic examination including cycloplegic refraction, age-appropriate visual acuity test, and binocular sensory testing where cooperation was sufficient. Cycloplegic RE was measured 30–45 min after instilling 1% cyclopentolate 2 times with 10 min intervals. Retinoscopy was applied 30 min after instillation of the last drop.

Best-corrected visual acuity was measured using the Snellen chart, Allen figures, or Lea Hyvarinen charts according to age groups. Stereopsis was assessed with random-dot stereopsis test. Sensory fusion was measured using the Worth-4-dot test at 5 m in cooperated children. If the patient reported seeing four dots, fusion was considered present. Suppression was recorded when the patients saw 3 points and diplopia was noted when they saw 5 points. Random-dot stereo test was evaluated as non-contoured (“randot”) and contoured (“circles and animals”) stereopsis. Contoured stereopsis was evaluated in two groups; as 20–50 s/arc (seconds of arc) and 70–400 s/arc, and all comparisons were performed based on this separation. Binocular sensory tests were performed under full cycloplegic hypermetropic correction. Near and distance deviation with and without refractive correction was measured by prism cover test in all cases. Ocular motility and convergence were also evaluated. Amblyopia was defined as a 2-line difference or more in interocular visual acuity. Visual acuity difference was calculated by converting Snellen visual acuity into logarithm of the minimum angle of resolution. Occlusion therapy was performed in cooperative patients.

### Potential Risk Factors Evaluated in the Study Group

- Age at onset of deviation
  Age at onset was determined according to the parent’s statement or the former ophthalmologist’s report. Analyses were evaluated in two age groups categorized as 0–6 months and older than 6 months.
- Duration of eye misalignment
  The duration of misalignment was determined by calculating the time interval between the onset of deviation and glass prescription. The period was categorized into two groups as 0–6 months and more than 6 months.
- The time interval between the age at onset of strabismus and the age of wearing glasses
  The period was categorized into two groups as 0–6 months and more than 6 months.
- Presence of anisometropia
  Anisometropia was defined as ≥1.0 D hypermetropic spherical equivalent (SE) RE difference between the two eyes.
- AC/A ratio
  This ratio was calculated with lens gradient method (2).
- High hyperopia and SE RE with cycloplegic examination
  Hyperopia was considered as high if it was ≥4.50 D SE.
- Amount of the uncorrected near and distance deviation at first visit.

Patients with AE were given maximum hyperopic correction to control the deviation, while patients with refractive AE (high AC/A ratio) were given bifocal lenses. The study excluded patients with partial AE and those who had surgery.

### Statistical Analysis

Statistical analysis was performed to determine the effect of these factors on the binocular visual outcomes, and data were analyzed by the Statistical Package for the Scientific Studies (SPSS) for Windows 25.0 (SPSS Inc., Chicago, IL). ^2^ and Fisher’s exact test, independent-t test, and Mann–Whitney U-test were used for the statistical analysis. A level of p<0.05 was considered statistically significant.

## Results

Patients who were diagnosed with AE between January 2010 and December 2019 at Baskent University Hospital, Department of Ophthalmology, were evaluated. Data from 139 patients were reviewed, but 107 patients were included in the study based on exclusion criteria. Demographic characteristics of the patients are shown in Table 1. Mean age at initial examination was 4.9±2.5 years (range: 1–12), and there were 52 girls. The mean follow-up was 34.3±28 months (range: 6–105). At first visit, the mean spherical RE was 4.07±2.00 D (1.00–9.75), and the mean SE was 4.29±2.00 D (1.00–9.00) in eyes with higher hyperopia. Fifty patients (46.7%) had a cycloplegic hypermetropic value of ≥4.50 D at least in one eye. The mean spherical RE was 3.61±1.90 D (0.25–8.25), and the mean SE was 4.07±2.00 D (1.00–9.25) in eyes with lower hyperopia. The initial mean deviation without correction was 30.4±13.5 PD (range: 6–60) at near and 24.67±23 PD (range: 0–55) at distance. High AC/A requiring bifocal glasses was present in 15.8% of the patients, inferior oblique overaction was present in 22.4%, and dissociated vertical deviation was found in 4.6%.

**Table 1.** Clinical characteristics of the study group

**Data of the study group (n=107)**	
Age of application to our clinic (years)	4.9±2.5 (1-12)
Gender	52 F, 55 M
Follow-up	34.3±28 months (6-105)
Age at onset of deviation	2±0.8 years (0-8)
Duration of application to doctor	0.3±0.3 years (0-3.2)
Duration of wearing on glasses	0.7±1.2 years (0-3.7)
Highest hypermetropic spherical value	4.07±2.00 D (1.00-9.75)
Highest hypermetropic SE	4.29±2.00 D (1.00-9.00)
Rate of > + 4.50 SE	46.7% (n=50)
Initial uncorrected near deviation	30.4±13.5 PD (6-60)
Initial uncorrected distance deviation	24.67±23 PD (0-55)
High AC/A ratio	15.8% (n=17)

F: Female; M: Male; PD: Prism diopter; D: Diopter; SE: Spherical equivalent. All values are defined as “mean±SD (range)”.

Table 2 summarizes the comparison between binocular sensorial outcome between the first and last visits of the patients. Visual acuity could be measured in 90% of the patients at the first visit. Random-dot stereopsis was statistically significantly different at final visit (p=001 for uncountered and p=0.01 for countered stereopsis). Amblyopia was determined in 37 patients (34.5%) at the first visit, and in 22 patients (20.5%) at last visit, which was statistically significantly different (p=0.001).

**Table 2.** Comparison of binocular sensorial outcome between the first and last visits

**Variables**	**Initial visit (%)**	**Final visit (%)**	**p-value**
Random-dot stereotests			
Uncontoured stereopsis	7.5	24.3	0.001^*^
Contoured stereopsis (20-50 sec/arc)	6.7	23.8	0.01^*^
Fusion	40.2	45.8	>0.05
Diplopia	14	20.6	>0.05
Suppression	19.6	7.5	>0.05
Amblyopia	34.5	20.5	0.001^*^

P-value was considered significant if <0.05. Statistical analysis was performed with Independent t-test.

In the comparative analysis of the demographics and clinical data of the patients with and without anisometropia, there were no significant differences in terms of age on application (p=0.69), age at onset of deviation (p=0.67), time interval until first examination (p=0.17), duration of wearing glasses (p=0.98), highest hyperopic SE (p=0.084), rate of SE >+ 4.50 D (p=0.076), initial uncorrected near deviation (p=0.41), initial uncorrected distance deviation (p=0.98), the rate of high AC/A ratio (p=0.74), and amblyopia (p=0.046). The hyperopic spherical RE was significantly higher in the anisometropic group (p=0.035). Amblyopia was identified in 50% of the patients with anisometropia while it was 29.1% in patients without anisometropia (p=0.046). When final binocular sensorial status was compared between the two groups, there was no statistically significant difference in terms of amblyopia (p=0.49), uncontoured stereopsis (p=0.69), contoured stereopsis (p=0.59), fusion (p=0.11), diplopia (p=0.84), and suppression (p=0.53).

A comparative analysis of the risk factors on final amblyopia, fusion, and stereopsis is shown in Table 3. There was a statistically significant relationship between the initial uncorrected distance deviation and amblyopia (p=0.041, independent t-test). However, Mann–Whitney U-test did not find significant relation (p=0.145).

**Table 3.** The analysis of the risk factors on final amblyopia, fusion, and stereopis

**Risk factors**	**Final amblyopia ratio**	**Final fusion ratio**	**Final stereopsis ratio**	
			**Uncontoured**	**Contoured (20-50 sec/arc)**
Age at onset of deviation				
<6 months	25.9%	44.4%	30%	30%
	p=0.759	p=0.24	p=0.279	p=0.12
>6 months	22.7%	62.9 %	50%	46.2%
Duration before application to doctor				
<6 months	25.6%	57.1%	46.2%	40%
	p=0.689	p=0.912	p=0.739	p=0.760
>6 months	21.4%	55. 6%	40%	45.5%
Duration of wearing glasses				
<6 months	26.9%	68.2%	47.4%	38.9%
	p=0.655	p=0.152	p=0.709	p=0.735
>6 months	22.2%	48.4%	41.2%	44.4%
Highest hypermetropic SE				
<4.50 D	19%	69.8%	32%	29.6%
	p=0.730	p=0.121	p=0.709	p=0.072
>4.50 D	22%	52.8%	41.2%	53.6%
Highest hypermetropic spherical RE	p=0.789	p=0.846	p=0.502	p=0.61
Initial uncorrected near deviation	p=0.90	p=074	p=0.058	p=0.011^*^
Initial uncorrected distance deviation	p=0.041^*^	p=0.021^*^	p=0.198	p=0.027^*^
		p=0.145^†^	p=0.072^†^		
AC/A ratio				
High	23.5%	64.3%	12.5%	11.1%
	p=0.741	p=0.848	p=0.049^*^	p=0.15
Low/Normal	20%	61.5%	50%	32%

P-value was considered significant if <0.05. SE: Spherical equivalent; D: Diopter; RE: Refractive error; AC/A: Accomodative convergance/accomodation. ^*^Indepentent t-test. ^†^Mann-Withney U test.

A significant correlation was found between uncorrected distance deviation and fusion according to the independent-t-test (p=0.021); however, no significance was found with the Mann–Whitney U-test (p=0.072). Other factors did not have a statistically significant effect on fusion. There was no significant relationship between the risk factors and random-dot stereopsis at final examination. Uncorrected near and distance deviation did not affect the random-dot test outcome. Random-dot stereo test was positive in 12.5% of patients with high AC/A and in 50% of patients without high AC/A (p=0.049). The correlation between these two groups could not be evaluated due to the small number of patients. Hyperopic SE, whether greater or less than 4.50 D, did not affect uncontoured stereopsis at final visit (p=0.709). In addition, when patients with SE greater and less than 4.50 D were compared, there was no significant difference between the two groups in terms of their relation to the time of onset of deviation, duration of deviation until the first examination, and first treatment (p=0.973, 0.843, and 0.455, respectively). Uncorrected near and distance deviations had significant effects on contoured stereopsis (p=0.011 and 0.027, respectively). In cases with mild near and distance deviation at first examination, contoured stereopsis with 20–50 s/arc was seen with a higher rate than contoured stereopsis with 70–400 s/arc.

## Discussion

In this cohort of patients with AE, we investigated the effects of age at onset of deviation, duration of misalignment, the time interval between the onset of strabismus and treatment, amblyopia, anisometropia, AC/A ratio, the amount of near and distant deviation at first visit, and cycloplegic hypermetropic and SE values on binocular sensory function. As a result, we found that uncorrected distance deviation at first visit had a significant effect on amblyopia, whereas the presence of anisometropia or a high AC/A ratio did not have a significant impact. When patients with or without anisometropia were compared, we found no difference in risk factors, but hypermetropic error was found to be significantly higher in the anisometropic group. There was no significant difference between the two groups in terms of stereopsis and fusion. Fusion was detected in 47.6% of the patients with anisometropia and 67.2% of the patients without anisometropia, but there was no statistically significant difference between these two groups.

The amblyopia rate was found to be 25.9% of patients diagnosed before 6 months of age and 40.9% of patients diagnosed after 6 months of age. However, no statistically significant difference was observed between the two groups. On contrary with the literature (6, 7), the rate of amblyopia was found to be significantly higher in patients with <6 months of wearing glasses compared to the patients who delayed wearing glasses. The presence of anisometropia was a significant risk factor for the development of amblyopia at the first visit but not for the final visit.

When examining patients with fusion and random-dot stereopsis at final visit, we found that only the uncorrected near and distant deviation had a significant effect on contoured stereopsis. While the effect of uncorrected distance deviation on amblyopia and fusion at last visit was significant with the independent t-test, it was not significant with the Mann–Whitney U-test. Therefore, this relationship does not seem to have a clear correlation. In literature, the effect of uncorrected near and distance deviation on binocular sensory outcome was evaluated only by Berk et al. and they found similar results (17).

Several studies have investigated the relationship between anisometropia and AE (6-10, 17-23). Anisometropia is frequently noted in association with AE, however, its potential role in the unsatisfactory outcome is under debate (6, 19-24). Birch et al. studied the factors that may influence the development of AE, such as family history, hypermetropic anisometropia, and abnormal binocular function (7). While 28% of the patients had anisometropia, 61% had esotropia. Family history, hyperopia >4.00 D, and anisometropia were found to be factors affecting the development of AE.

In a study conducted by Fawcett et al. on 69 patients, risk factors for abnormal binocular vision were evaluated after good alignment of AE, and anisometropia was not found to pose a significant risk for abnormal binocular vision (10). In our study, although anisometropia was found in one-quarter of all patients, no significant relationship was found with binocular sensory outcomes, similar to the aforementioned study. Weakley et al. observed that in patients with mean SE of +3.00 D or more, anisometropia increased the risk of developing AE and esotropia (6). They reported that the high AC/A ratio was similar in the anisometropic and non-anisometropic groups and had no effect on the binocular visual outcome, consistent with our results. On the contrary, we found that the SE values were higher in the anisometropic group than in the non-anisometropic group.

Fawcett et al. found that the age of onset of deviation was not a risk factor for abnormal binocular function and did not find a difference in this respect between infantile and childhood esotropia (10). The study also investigated the duration of uncorrected deviation as a risk factor for abnormal binocular function. They found that the abnormal stereopsis rate was 4.6 times higher and the abnormal fusion rate was 31 times higher in the late treatment group. In our study, we did not find any difference between patients whose age at onset of deviation was before and after 6 months in terms of this relationship. We did not see any difference in binocular function between the early treated group and 6 months late treated group. Coats et al. evaluated 17 cases of esotropia who were diagnosed before the age of one and showed that refractive AE was diagnosed as early as 4 months of age (16). In the early-onset AE group, amblyopia was not observed with treatment, and long-term success was achieved with full hypermetropic correction.

In the study of Berk et al. evaluating the clinical and functional results of AE, amblyopia was found in 87 of 147 (59.2%) patients at first examination, and anisometropia was found to be the only significant risk factor (17). Similar to our results, a better fusion but worse stereopsis was found after treatment. In early-onset esotropia, both cycloplegic refraction values and deviation angle were found to be higher, but the relationship was not statistically significant. In our study, we could not find any relation between the age of onset of esotropia (before or after 6 months) and the hypermetropic values at first visit. We did not find any difference between the late- and early-onset deviation groups in terms of the incidence of amblyopia and binocular function at final visit. In patients without amblyopia, the time interval between the onset of deviation and the treatment was shorter, and the mean cycloplegic refraction was lower. However, the relationship was not significant.

Uretmen et al. studied the factors affecting stereoacuity in refractive AE patients and demonstrated that despite successful optical correction, 50% of patients had an abnormal binocular sensory function, and the only risk factors were determined as intermittent or constant misalignment (19). These findings may indicate early and appropriate optical correction to minimize the adverse effects of ocular misalignment on binocular sensory function. In the outcome of our study, we achieved random-dot stereopsis in only 24.3% of the patients and fusion in 45.8% at final visit. In contrast, Mulvihill et al. found the rate of stereopsis as high as 89.3% at final visit in a similar study (18).

There are inherent limitations in a retrospective chart review. Some cases were followed up at an outside facility and had later ophthalmic referral to us. The patient data including previous occlusion therapy or the age at onset of deviation and the duration of deviation before strabismus surgery were obtained from the parents. It would be more meaningful if we could evaluate the patients starting from their initial complaints.

## Conclusion

In conclusion, anisometropia, age of onset and duration of eye misalignment before treatment, and high hyperopia and high AC/A ratio did not affect binocular function in children with AE. Uncorrected distance deviation at first examination was identified as a risk factor for abnormal binocular vision. In addition, uncorrected near deviation affected contoured stereopsis at final examination. Assessment of these risk factors can help identify patients most likely to benefit from treatment for binocular sensory function.

## Disclosures

### Ethics Committee Approval:

Baskent University Ethics Committee, protocol number: E-94603339-604.01.02-2512, Date: 13/01/2021.

### Peer-review:

Externally peer-reviewed.

### Conflict of Interest:

None declared.

### Authorship Contributions:

Involved in design and conduct of the study (SAB, SO, SA, AP); preparation and review of the study (SAB, SO, ZO, BU); data collection (SAB, SO, ZO, BU, AP); and statistical analysis (SA, SAB).
